# Detection and Phylogenetic Analysis of *Wolbachia* in the Asiatic Rice Leafroller, *Cnaphalocrocis medinalis*, in Chinese Populations

**DOI:** 10.1673/031.011.12301

**Published:** 2011-09-19

**Authors:** Huan-Na Chai, Yu-Zhou Du, Bao-Li Qiu, Bao-Ping Zhai

**Affiliations:** ^1^ Institute of Applied Entomology, Yangzhou University, Yangzhou 225009, China; ^2^ Department of Entomology, South China Agricultural University; ^3^ Department of Entomology, Nanjing Agricultural University, Nanjing 210095, China

**Keywords:** infection rate, phylogenetic relationship

## Abstract

*Wolbachia* are a group of intracellular inherited endosymbiontic bacteria infecting a wide range of insects. In this study the infection status of *Wolbachia* (Rickettsiales: Rickettsiaceae) was measured in the Asiatic rice leafroller, *Cnaphalocrocis medinalis* (Guenée) (Lepidoptera: Pyralidae), from twenty locations in China by sequencing *wsp, ftsZ and* 16S rDNA genes. The results showed high infection rates of *Wolbachia* in *C. medinalis* populations. *Wolbachia* was detected in all geographically separate populations; the average infection rate was ∼ 62.5%, and the highest rates were 90% in Wenzhou and Yangzhou populations. The *Wolbachia* detected in different *C. medinalis* populations were 100% identical to each other when *wsp, ftsZ,* and 16S rDNA sequences were compared, with all sequences belonging to the *Wolbachia* B supergroup. Based on *wsp, ftsZ* and 16S rDNA sequences of *Wolbachia,* three phylogenetic trees of similar pattern emerged. This analysis indicated the possibility of inter-species and intra-species horizontal transmission of *Wolbachia* in different arthropods in related geographical regions. The migration route of *C. medinalis* in mainland China was also discussed since large differentiation had been found between the *wsp* sequences of Chinese and Thai populations.

## Introduction

The genus *Wolbachia* (Rickettsiales: Rickettsiaceae) is a group of intracellular gram-negative and vertically inherited endosymbiontic bacteria that belong to the order Rickettsiales in the α-subdivision of the class Proteobacteria (Werren 1997). Numerous surveys indicate that *Wolbachia* can infect a wide range of arthropods and filarial nematodes. Recent surveys indicate that 20% to 76% of examined insects harbor *Wolbachia* (Hilgenboecker et al. 2008), as well as many arachnids, terrestrial crustaceans, and mites, making this group one of the most widespread obligate bacterial endosymbionts ever described (Cordaux et al. 2001; Gotoh et al. 2003; Rowley et al. 2004).

*Wolbachia* play important roles in ecology, evolution, and reproductive regulation in their hosts (Werren 1997), and have been considered as a potent evolutionary force. In nematodes, *Wolbachia* appear to play a mutualistic role in development and reproduction (Bandi et al. 1999; Langworthy et al. 2000; Bandi et al. 2001; Casiraghi et al. 2002). In arthropods, *Wolbachia* often obligatorily live inside the cytoplasm in reproductive tissues and are associated with a number of different reproductive phenotypes in its hosts, such as cytoplasmic incompatibility (Shoemaker et al. 1999; Hurst and Werren 2001; Bordenstein and Wernegreen 2004; Jaenike et al. 2006), feminization, parthenogenesis inducing, male killing, and modifying fecundity (Hurst et al. 1999; Stouthamer et al. 1999; Baldo et al. 2005). These reproductive symptoms are regarded as selfish strategies of the symbionts whereby the frequency of female offspring increased (O'Neill et al. 1997; Werren 1997; Stouthamer et al. 1999; Haine 2008; Werren et al. 2008). Some *Wolbachia* strains also reduce host fitness by reducing fecundity or modifying growth rates (Chu et al. 2005), although the mechanism is not well understood.

*Wolbachia* cannot be cultured outside their hosts, so detection of infection has been based largely on amplification of *Wolbachia* DNA using allele-specific polymerase chain reactions (PCR). To date, *wsp,* 16S rDNA, *ftsZ, groEL, coxA, fbpA, hcpA, gatB, dnaA* and *gltA* genes have all been characterized and used for phylogenetic studies. *Wolbachia* strains are usually clustered into eight divergent clades based on these genes, which are described as supergroups A–H (O'Neill et al. 1992; Bandi et al. 1998; Zhou et al. 1998; Schulenburg et al. 2000; Werren and Windsor 2000; Lo et al. 2002; Bordenstein and Rosengaus 2005; Casiraghi et al. 2005) and IK (Gorham et al. 2003; Casiraghi et al. 2005; Ros et al. 2009), which were added later.

The Asiatic rice leafroller, *Cnaphalocrocis medinalis* (Guenée) (Lepidoptera: Pyralidae), is a migratory rice pest with 1–11 generations depending on its geographical distribution in rice-planting regions worldwide. *C. medinalis* also distributes widely in rice production areas from north to south in China, from Heilongjiang province and Inner Mongolia autonomous region to Taiwan and Hainan, excluding the Xinjiang and Ningxia autonomous region. In recent decades, *C. medinalis* has caused serious decreases in rice yields in China most notably due its outbreak from 2003–2005 (Liu et al. 2008).

In this study, the infection status of the endosymbiont *Wolbachia* in *C. medinalis* from twenty different regions of China was determined, and the genetic differentiation between the *Wolbachia* strains from Thailand and China were also analyzed. Study of the infection status and transmission mechanism of *Wolbachia* has been considered to be very helpful in utilization of insect natural enemies for pest control.

## Materials and Methods

### Insect sample collection

In July and October 2009, the samples of *C. medinalis* larvae were collected from 20 paddy fields in 15 provinces of China (Table 2). When sampling, *C. medinalis* 1^st^–4^th^ instar larvae were collected, placed in 95% ethanol, marked with the location and time, and taken back to the laboratory for further analysis.

### DNA extraction

To extract DNA, the entire body was used if the larva was < 1 mm in length, while only the abdomen was used if larva size was ∼ 1–5 mm. DNA was extracted according to the description of Ahmed et al. (2009). Briefly, samples were washed several times by double distilled water and put into 1.5 ml centrifuge tubes with DNA extraction buffer (100 mmol/L Tris-HCl, pH 8.0, 50 mmol/L NaCl, 50 mmol/L EDTA, 1% SDS, 0.15 mmol/L Spermine, 0.5 mmol/L Spermidine) and proteinase K (20 mg/mL). Samples were homogenated and digested at 56° C for three hours. The homogenate was mixed afterwards with an equal volume of phenol for 10 minutes and centrifuged at 12,000 rpm for four minutes. The centrifugation was repeated twice, and chloroform (isoamyl alcohol 24:1) was used instead of phenol for the last repetition. The supernatant was precipitated overnight at -20° C and then centrifuged at 12,000 rpm for 20 minutes to sediment the DNA pellet. The pellet was then allowed to dry at room temperature. The dried DNA pellet was re-suspended in 30 µl of TE buffer (10 mM Tris-HCl, 1 mM EDTA, pH 8.0) and kept at 4° C until use for PCR.

Twenty larvae from each *C. medinalis* population were selected randomly for DNA extraction and further detection.

### PCR amplification

Three pairs of primers were used to amplify the *wsp, ftsZ* and 16S rDNA fragments of different *C. medinalis* samples by PCR, according to the methods of Braig et al. (1998), Werren et al. (1995), and West et al. (1998). Polymerase chain reactions were done in 20 µl reaction volumes containing: 2 µl 10× PCR buffer (TakKara Bio, www.takarabio.com), 2 µl 25 mM MgCl_2_, 1.5 µl dNTPs (10 mM each), 1.0 µl forward primer, 20 µl reverse primer, and 1.2 units of Taq DNA polymerase (Takara). To achieve the final volume of 20 µl, double distilled H2O was added. For *Wolbachia wsp* gene amplification, the primers were *wsp*-F: 5′ TGGTCCAATAAGTGAGAGAAAC 3′ and *wsp*-R: 5′AAAAATTAAACGCTACTCCA 3′. The *Wolbachia ftsZ* PCR primers were *ftsZ*-F: 5′TACTGACTGTTGGAGTTGTAACTAAGC CGT 3′ and *ftsZ*-R: 5′-TGCCAG TTGAAGAAACTCTAACTC 3′; both the *wsp* and *ftsZ* primers can amplify a 0.6 kb DNA fragment (Zhou et al. 1998; Jeyaprakash and Hoy 2000). The primers for *Wolbachia* 16S rDNA PCR amplification were 16S-F: 5′ TTGTAGCCTGCTATGGTATAACT 3′ and 16S-R: 5′ GAATAGGAGTTTTCATGT 3′, amplifying a 0.9 kb DNA fragment (O'Neill et al. 1992).

The PCR amplification program of *wsp* and 16S rDNA primers included an initial denaturation at 94° C for three minutes followed by 35 cycles with a denaturation step at 94° C for one minute, annealing at 55° C for one minute, extension at 72° C for two minutes, and final extension at 72° C for 10 minutes. The *ftsZ* PCR amplification program was done according to three linked profiles. First, one cycle of denaturation at 94° C for three minutes followed by 10 cycles of denaturation at 94° C for 10 seconds, annealing at 65° C for 30 seconds, and extension at 68° C for one minute. This was followed by 25 cycles, each cycle with a denaturation step at 94° C for 10 seconds, annealing at 65° C for 30 seconds, extension at 68° C for one minute, plus an additional 20 seconds for each consecutive cycle. All PCR amplifications were done in a Thermal Cycler 48 (Bioer Technology, www.bioer.com.cn).

### PCR Product Detection, Cloning, Sequencing and Analysis.

The amplified PCR products were electrophoresized on 1.0% agarose gel with water as a negative control, then cloned, screened, and two-way sequenced on an ABI PRISMTM 3730XL Automated DNA Sequencer (Applied Biosystems, www.appliedbiosystems.com). Six positive clones were sequenced per insect.

The *wsp, ftsZ,* and 16S rDNA sequences of *Wolbachia* from different *C. medinalis* populations were first blasted in NCBI, then analyzed and aligned with DNAStar (www.dnastar.com) and Clustal X1.83 (www.clustal.org). Some related *Wolbachia wsp, ftsZ,* and 16S rDNA sequences in other insects such as whitefly *Bemisia tabaci* and butterfly *Pieris rapae* were downloaded as references (Table 1). ANOVA was used to identify and compare differences among populations and all data was analyzed using DPS software (Tang and Feng 2002). Phylogenetic trees were constructed using Mega 4.0 software (MEGA, www.megasoftware.net) with maximum parsimony and neighbor joining methods (maximum likelihood model). Bootstrap analysis was done with 1000 replications, and bootstrap values were calculated using a 50% majority rule.

**Table 1.**
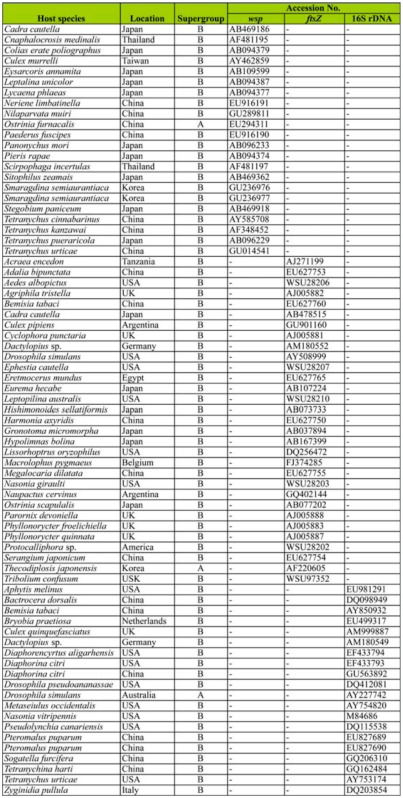
Reference sequences of *Wolbachia* used in the phylogenetic analyses.

**Table 2.**
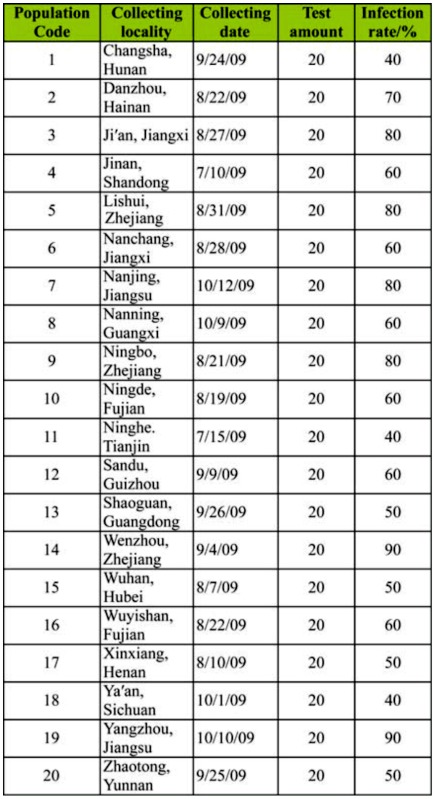
*Cnaphalocrocis medinalis* samples collected in China for *Wolbachia* detection.

Some reference sequences of *Wolbachia* from different insects as well as mites were downloaded from GenBank (Table 1) for *Wolbachia* phylogenetic analysis. Three reference sequences belonging to the *Wolbachia* A supergroup were used as the outgroup in the phylogenetic trees of *wsp,* *ftsZ*, and 16S rDNA; *Ostrinia furnacalis* (EU294311), *Thecodiplosis japonensis* (AF220605), and *Drosophila simulans* (AY227742).

## Results

### *Wolbachia* detection in *C. medinalis* by PCR

**Figure 1.**
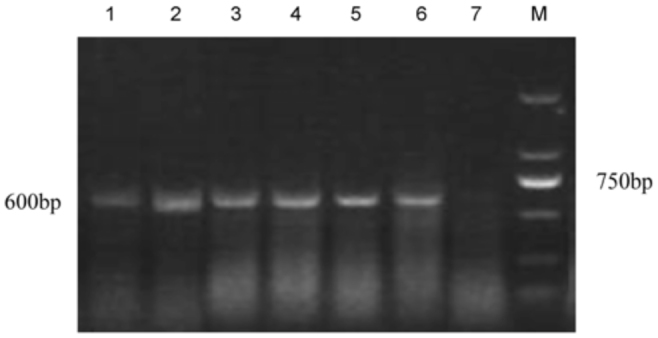
Electrophoresis of PCR products of *Wolbachia wsp* gene from *Cnaphalocrocis medinalis* by general primers. M: Molecular size standards, lane 7: negative control, lanes 1–6: different regions of *C. medinalis* (corresponding population number 1 to 6 in Table 2). High quality figures are available online.

**Figure 2.**
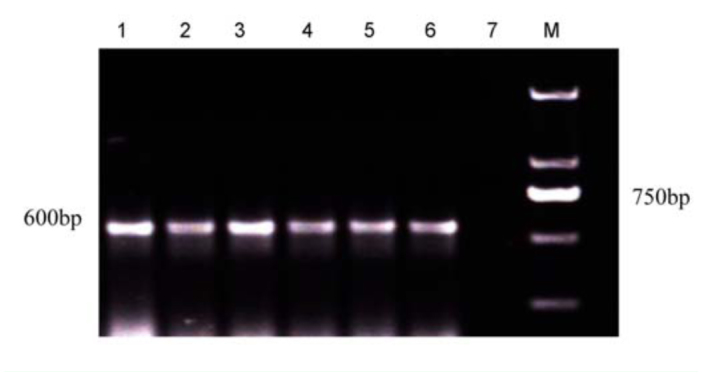
Electrophoresis of PCR products of *Wolbachia ftsZ* gene from *Cnaphalocrocis medinalis* by special primers. M: Molecular size standards, lane 7: negative control, lanes 1–6: different populations of *Cnaphalocrocis medinalis* (corresponding population code 1 to 6 in Table 2). High quality figures are available online.

**Figure 3.**
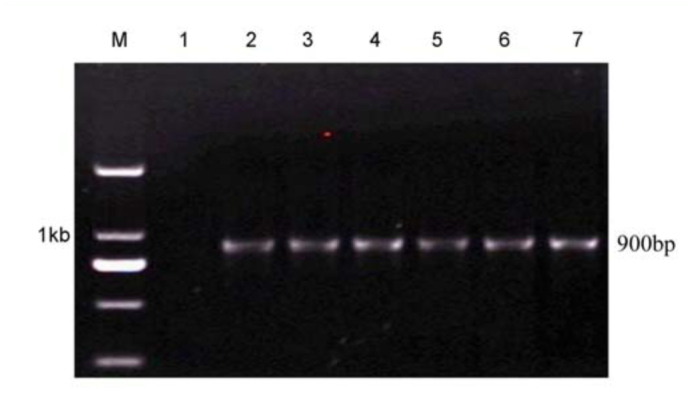
Electrophoresis of PCR products of *Wolbachia* 16S rDNA gene from *Cnaphalocrocis medinalis* by special primers. M: Molecular size standards, lane 1: negative control, lanes 2–7: different populations of *C. medinalis* (corresponding population code 1 to 6 in Table 2). High quality figures are available online.

The infection of *Wolbachia* in different *C. medinalis* populations was detected by PCR with *wsp, ftsZ,* and 16S rDNA genes. Results showed that in total, 250 of 400 individual larvae from 20 populations were detected to be positive. The *wsp* and *ftsZ* PCR products were ∼ 600 bp (Figures 1 and 2) while the 16S rDNA PCR products of *Wolbachia* from *C. medinalis* were ∼ 900 bp (Figure 3). The align results with DNAStar software indicated that all the *wsp* sequences of *Wolbachia* from 250 *C. medinalis* individuals were 100% identical to each other. Similar results were obtained from the *ftsZ* sequences and 16S rDNA sequences.

### The infection rate of *Wolbachia* in different *C. medinalis* populations

The infection rates of *Wolbachia* in the twenty *C. medinalis* populations collected from different geographical regions varied significantly (*F* = 2.3750, *p* < 0.01, df = 19), ranging from 40–90% with an average of 62.5% (Table 2). The highest infection rates were found in the *C. medinalis* Yangzhou and Wenzhou populations (90%), while the lowest rates were recorded in Ya' an, Changsha, and Tianjin populations (40%).

### Phylogenetic analysis of the *Wolbachia wsp, ftsZ,* and 16S rDNA sequences

**Figure 4.**
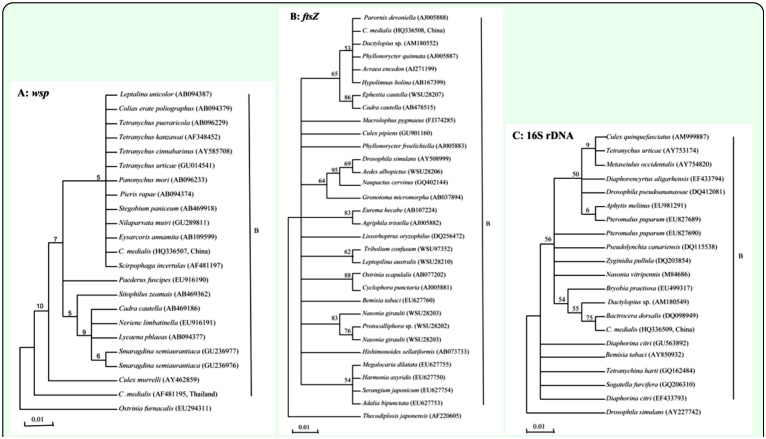
The phylogenetic trees of *Wolbachia* detected in *Cnaphalocrocis medinalis* and other insects and mites based on their *wsp*, *ftsZ,* 16S rDNA sequences. Trees inferred from maximum parsimony and neighbor joining methods (maximum likelihood model) using MEGA 4.0 program were similar though less resolved (data not shown.) The sequences of *Ostrinia furnacalis* (EU2943 11) and *Thecodiplosis japonensis* (AF220605), were used as outgroups in Fig. 4A and B, respectively. Additionally, the sequence of *Drosophila simulons* (AY227742) was used in Fig.4C. High quality figures are available online.

The phylogenetic analysis results of the *Wolbachia wsp* sequences from *C. medinalis* and other 21 reference sequences are shown in Figure 4a. There were two major branches in the phylogenetic tree based on *Wolbachia wsp* sequences (HQ336507). The first branch clustered *Wolbachia* sequences from the *C. medialis* Chinese population and the *Scirpophaga incertulas* (AF481197) Thailand population, containing 11 *Wolbachia wsp* genes of insects and mites from Japan, China, Korea, and Thailand, into a single clade. The second branch included six *Wolbachia* populations from five species of insects including *Sitophilus zeamais* (AB469362), *Cadra cautella* (AB469186), *Neriene limbatinella* (EU916191), *Lycaena phlaeas* (AB094377), and *Smaragdina semiaurantiaca* (GU236976-7). This second branch, while comprised of insects from different taxonomic families and orders, served as a sister branch with *Paederus fuscipes* (EU916190) and the first branch. However, one reference sequence of *Wolbachia wsp* gene from *C. medialis* Thai population has been found to be clustered in the periphery except near outgroup in the tree; the homology of the *Wolbachia wsp* sequences from *C. medialis* Chinese and Thai populations was only 83.2%.

Figure 4b showed the phylogenetic analysis results based on the *ftsZ* gene sequnces of *Wolabchia* in *C. medinalis* and other hosts. Result revealed that the *ftsZ* sequence (HQ336508) in *C. medinalis* samples shared 100% identity with those sequences from *Acraea encedon* (AJ271199), *Hypolimnas bolina* (AB167399), *Phyllonorycter quinnata* (AJ005887) and *Parornix devoniella* (AJ005888). Four reference sequences from four species of lady beetles (EU627750, EU27753-5) clustered into one clade, showing 100% identity to each other and the lowest homology to the sequence from *C. medinalis.* The homology of all sequences of the *Wolbachia ftsZ* gene from the rice leaf roller and lady beetles was close to identical at 98.9%.

The phylogenetic tree of 16S rDNA sequences of *Wolbachia* was shown in Figure 4c. Similar to the *wsp* and *ftsZ* tree, no regular pattern was found in this tree. The 16S rDNA sequences (HQ336509) of *Wolbachia* from *C. medinalis* was first clustered into a subclade with *Bactrocera dorsalis* (DQ098949), sharing 99% identity, then subsequently was clustered into higher clades with other sequences from different hosts in various families and orders.

## Discussion

Since first discovered in *Culex pipiens* (Hertig 1936), *Wolbachia* have been described as a widespread and common insect bacteria all over Neotropical (Borm et al. 2003), Palaearctic (West et al. 1998) and Nearctic regions (Werren and Windsor 2000). Samples of insect species from these three regions have almost the same *Wolbachia* infection rate (20%). Over 50% of a set of Southeast Asian ant species tested positive for *Wolbachia* (Wenseleers et al. 1998), Jeyaprakash and Hoy (2000) found over 76% of the samples of insect species were infected with *Wolbachia,* and in the current study, the average infection rate of *Wolbachia* in the Asiatic leafroller in China was 62.5%, ranging from 40 to 90% depending on their geographical distributions. Such findings support that *Wolbachia* is wide spread in numerous arthropods.

*Wolbachia* are mostly transmitted through egg cytoplasm of the hosts from parents to offspring, but several studies revealed that they did not have a consistent relationship between *Wolbachia* and hosts in phylogenetic trees (Schilthuizen and Stouthamer 1998; Zhou et al. 1998). It is supposed that there may be horizontal transmission between different hosts, including intra-species and inter-species transmission (Heath et al. 1999). This phenomenon has proven very common in spiders (Rowley et al. 2004) as well as between some arthropods and their parasitoids (Jeyaprakash and Hoy 2000), though the mechanism of this horizontal transmission is still not clear. For example, the horizontal transmission of *Wolbachia* has been found in a parastic wasp, *Nasonia giraulti,* and their blowfly hosts *Protocalliphora* sp., in some drosophilid parasitoids and their hosts, as well as in the parasitoid *Leptopilina boulardi* and their fly host *D. simulans* (Werren et al. 1995; Heath et al. 1999; Vavre et al. 1999). In our study, no regular pattern was found in any of the three phylogenetic trees for the genetic relationship between *C. medinalis* and other insects or mites; the *Wolbachia* sequences from *C. medinalis* clustered into one clade with different insects or mites in different trees. This suggests two conclusions. First, while there was no direct evidence to verify the horizontal transmission of *Wolbachia* between their hosts, the pattern of transmission of *Wolbachia* was not limited within host species and by geographical locations. Second, as the *Wolbachia* from *C. medinalis* Chinese population was clustered into one clade with different insects or mites in different trees, it was not possible to determine the transmission among arthropods in the field by comparing one phylogenetic tree using single genes.

In the current study, all the *Wolbachia* sequences from the different geographical populations were 100% identical to each other, which suggested that no evolutionary differentiation had occurred in China. This may have been due to the migration of *C. medinalis* since it is a migratory pest. For example, during population expansion, the *Wolbachia—infected* males and females could have spread widely in China, thus increasing the opportunities of *Wolbachia* being transmitted through a broader range by mating between or among individuals from the same or different regions. For example, Huigens et al. (2000 and 2004) found frequent horizontal transmission from infected to uninfected wasp larvae that shared a common food source. Frequent horizontal transmission occurred between infected and uninfected *Trichogramma kaykai, Trichogramma deion, Trichogramma pretiosurm, Trichogramma atopovirilia* when eggs were laid in their common host *Apodemia mormodeserti*. The transferred *Wolbachia* were then vertically transmitted to the new host's offspring. However, this aspect needs further research to reveal this complex mechanism.

Kittayapong et al. (2003) used Long PCR and long *wsp* primers to investigate the infection status of *Wolbachia* in *C. medinalis* collected from rice fields in 29 provinces of Thailand. They showed that the average infection rate in the *C. medinalis* Thai populations was 48.8%, which was much lower than the infection rate in our study. This difference is possibly related to sample size. Similar to those found in China, all the detected *Wolbachia* sequences using *wsp* gene in 29 provinces of Thailand were 100% identical and belong to the *Wolbachia* B supergroup. However, large differentiation between the *Wolbachia wsp* sequences from *C. medinalis* Chinese and Thai populaions was found; sequence homology was 83.2%. It is thought that the *C. medinalis* population in China may have come from Southeast Asia and entered into China by the first northward mass migration during early March to April. Additionally, *C. medinalis* populations from different regions of China may be those migrants from Thailand or their offspring. Thus, *Wolbachia* in *C. medinalis* Chinese and Thai populations should be the same strain or quite close to each other (Chang et al. 1980; Gao et al. 2008). However, the results in our study showed large differences between the *Wolbachia wsp* sequences in the *C. medinalis* Chinese and Thai populations. We speculate that either the first northward mass migration of *C. medinalis* was not from the direction of Thailand, or that *Wolbachia* in Chinese populations may have displaced the strain of *Wolbachia* in Thailand populations when *C. medinalis* migrated to China from Thailand. Further investigation in this area is needed.

In summary, the infection status of *Wolbachia* in *C. medinalis* Chinese populations was investigated and the average infection rate of *Wolbachia* was found to be 62.5%. The phylogenetic trees based on the *wsp, fstZ,* and 16S rDNA revealed the possibility of interand intra-species horizontal transmission of *Wolbachia* in different arthropods. In view of the biological roles of *Wolbachia* in their hosts, especially for host reproduction such as cytoplasmic incompatibility and male killing, further studies on how to make good use of the transmission patterns of *Wolbachia* to enhance the biological control of pests deserves more emphasis (Gong and Shen 2002; Tsai et al. 2002; Miao et al. 2004; Zabalou et al. 2004; Pfarr and Hoerauf, 2005; Ruan and Liu 2005).
